# Electrophoretic Particle Guidance Significantly Enhances Olfactory Drug Delivery: A Feasibility Study

**DOI:** 10.1371/journal.pone.0086593

**Published:** 2014-01-31

**Authors:** Jinxiang Xi, Xiuhua A. Si, Rachel Gaide

**Affiliations:** 1 Department of Mechanical and Biomedical Engineering, Central Michigan University, Mount Pleasant, Michigan, United States of America; 2 Department of Engineering, Calvin College, Grand Rapids, Michigan, United States of America; 3 Department of Engineering, Calvin College, Grand Rapids, Michigan, United States of America; Université Lyon, France

## Abstract

**Background:**

Intranasal olfactory drug delivery provides a non-invasive method that bypasses the Blood-Brain-Barrier and directly delivers medication to the brain and spinal cord. However, a device designed specifically for olfactory delivery has not yet been found.

**Methods:**

In this study, a new delivery method was proposed that utilized electrophoretic forces to guide drug particles to the olfactory region. The feasibility of this method was numerically evaluated in both idealized 2-D and anatomically accurate 3-D nose models. The influence of nasal airflow, electrode strength, and drug release position were also studied on the olfactory delivery efficiency.

**Findings:**

Results showed that by applying electrophoretic forces, the dosage to the olfactory region was significantly enhanced. In both 2-D and 3-D cases, electrophoretic-guided delivery achieved olfactory dosages nearly two orders of magnitude higher than that without electrophoretic forces. Furthermore, releasing drugs into the upper half of the nostril (i.e., partial release) led to olfactory dosages two times higher than releasing drugs over the entire area of the nostril. By combining the advantages of pointed drug release and appropriate electrophoretic guidance, olfactory dosages of more than 90% were observed as compared to the extremely low olfactory dosage (<1%) with conventional inhaler devices.

**Conclusion:**

Results of this study have important implications in developing personalized olfactory delivery protocols for the treatment of neurological disorders. Moreover, a high sensitivity of olfactory dosage was observed in relation to different pointed release positions, indicating the importance of precise particle guidance for effective olfactory delivery.

## Introduction

**Figure 1:**
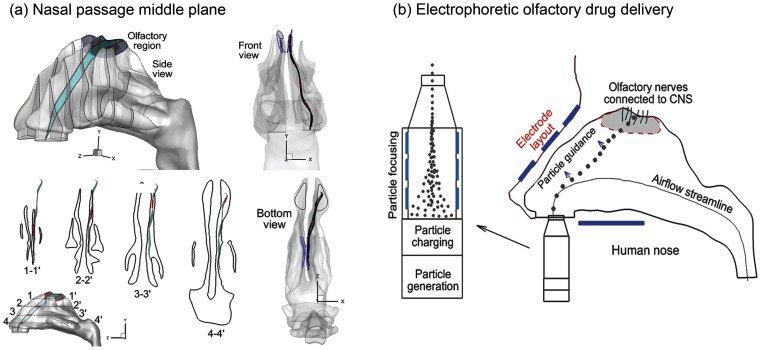
Nasal airway anatomy with the olfactory region (OR). Geometric complexity and narrowness of the nasal airway prevents effective intranasal drug delivery. To avoid contact loss to the wall, particles should closely follow the middle plane (a) of the nasal passage, which exhibits three-dimensional spatial features. A simple delivery system is shown in (b) which consists of components with the following four functions: (1) aerosol generation (inhaler), (2) particle charging, (3) particle focusing, and (4) particle navigation control. A more elaborate system can have supports to stabilize the device relative to the human head.

The Blood-Brain-Barrier (BBB) has forestalled the use of many new genetically engineered drugs for treating central nervous system (CNS) disorders such as Alzheimer’s disease [1], paraplegia [2], and migraine headache [3]. The need for developing a simple, safe, and effective way of delivering drugs for the treatment of CNS disorders is urgent. The olfactory region is the only site in the human body where the CNS is in direct contact with the outer environment. If drugs could be directly sent to this region through the nose, they can diffuse through the olfactory mucosa and reach the CNS via the extracellular epithelial pathway [4]. Direct nose-to-brain drug delivery circumvents the BBB and has multiple advantages over conventional intravenous delivery methods. These advantages include ease of administration, rapid onset of action, and avoidance of first-pass metabolism [5], [6]. However, demonstration of its clinical feasibility is still in adolescence due to a lack of devices that effectively deliver medication to the olfactory region. While there are many nasal delivery devices available on the market such as a nebulizer, powder inhaler, or pressurized metered-dosed inhaler (pMDI), devices designed for olfactory deposition have not yet been found [7]. The limitations of conventional nasal devices are obvious; only a very small fraction of therapeutic agents deposit in the olfactory region and enter the brain. Previous studies [8], [9] have shown that less than 1% of nasal-inhaled particles could reach the olfactory nerves which are secluded in the upmost part of the nasal cavity (Fig. 1a). Therefore, it is critical to search for more effective methods to deliver drugs to the olfactory region.

The low olfactory delivery efficiency is mainly attributed to the unique structure of the human nasal cavity, which consists of two relatively symmetric nasal passages separated by a nasal septum. Each nasal passage has three curved fin-like airway protrusions known as the superior, middle and inferior meatus. The osseous tissues above each meatus are termed the superior, middle, and inferior turbinates, respectively, and form the lateral wall of the main passage. The olfactory epithelium, or olfactory mucosa, is located at the very top of the nasal cavity between the superior turbinate and the cribriform plate of the ethmoid bone (Figs. 1a) and covers 10∼12 cm^2^, or approximately 8% of the total nasal surface area [10]. If drugs can be delivered deep and high enough into the nasal cavity, they will reach the olfactory mucosa and enter the brain. However, the intricacy of the nasal geometry prevents effective intranasal drug delivery. The particle motions are highly complex and far from planar, as shown in Fig. 1a. Most of the inhaled particles will impinge on the nasal walls and be filtered out. Conventional inhalation drug delivery devices depend solely on inhalation aerodynamics to direct drug particles to the target site [11]. The three major forces acting on these particles are inertia, drag force, and gravity. There is no further control on the trajectories of these particles once they have been released from an inhalation device. Accordingly, how far a particle travels, or where it deposits primarily depends on its initial velocity as well as the nasal airway structure. Due to this lack of control, a significant amount of medication is wasted in the upper airway and cannot reach the targeted olfactory receptor.

The rationale of electrophoretic drug delivery can be compared to that of guided ballistic missiles. The missiles can precisely hit their targets because they are under continuous control along specified trajectories. Similarly, it would be highly desirable if we could control the pharmaceutical particles so that they behave much like guided missiles. This seems challenging considering the tiny size of these aerosols (0.2–5 microns) and the complex nasal structure, which requires extreme accuracy to navigate the particles through the convoluted conchae and toward the hidden olfactory region. Interestingly, the solution to this problem lies in the problem itself, or the tiny particle size. Electrophoretic force on nanoparticles can be dominate, which make it possible to remotely control inhaled aerosols. The smaller the size of the particles, the more they are influenced by the electromagnetic force [12]. It is hypothesized that with an appropriate external electric field, charged nanoparticles can be precisely guided via electrophoretic forces and maneuvered through the nasal passages without loss to the airway walls. The required electric field can be achieved by carefully arranging multiple electrodes with different electric potentials around the nose.

Charged particles have found their way into many industrial and medical applications, such as ink jet printing, electrostatic precipitation, and mass spectrometry. The sizing, classification, and collection of nanoparticles primarily depends on their electrical properties [13]. In light of inhaled charge particles, a number of theoretical and experimental studies have considered the transport and deposition of charged particles in human respiratory tract [14], [15], [16], [17], [18], [19], [20], [21]. By gradually reducing the particle charges in a nasal replica cast, Fry [19] reported that partially neutralized particles and Boltzmann charged particles had similar deposition characteristics in the nasal cavity. However, the charge levels in this study are well below those typical of metered dose inhalers that are sufficiently high to alter deposition of inhaled particles in the respiratory tract [17]. Boltzmann charge on submicron aerosols can be significant [13] and may cause some level of deposition variations that were not identified by Fry [19]. Considerable deposition enhancement has been reported in the larynx and tracheobronchial cast for both micrometer [14] and submicron [15] charged particles, even in the absence of an external electric field. Yu and coworkers [18], [22] theoretically studied the electrostatic precipitation in pipe flows with specified electric potentials and proposed empirical correlations for charged aerosol depositions in such idealized geometries. However, these correlations, which were based on fully developed flows, cannot be readily extended to complex geometries such as the respiratory tract where developing flow prevails.

The objectives of this study are to (1) develop a computational fluid dynamics (CFD) model of electrophoretic-guided drug delivery; (2) numerically evaluate the feasibility of electrophoretic focusing in three quadrupole designs; (3) numerically evaluate the efficiency of the electrophoretic-guided drug delivery in both idealized 2-D and physiologically realistic 3-D nose models; and (4) identify the optimal layout and operating parameters of the electrodes. Potential benefits from this study include (1) insight into the transport dynamics underlying the delivery of intranasal neurological medications and (2) guidelines for the development of effective drug delivery devices that target the olfactory epithelium.

## Methods

### Nasal Airway Model

A physiologically accurate airway model is necessary for reliable analysis of inhalation drug delivery. Image-based modeling represents a remarkable improvement over conventional cadaver castings that are subject to large distortions due to the shrinkage of mucous membranes or insertion of casting materials. MRI scans of a healthy non-smoking 53-year-old male (weight 73 kg and height 173 cm) were used in this study to construct the nasal airway model (Fig. 1a). The MRI tracings were provided by the Hamner Institutes for Health Sciences (Research Triangle Park, NC), the use of which has been approved by the Virginia Commonwealth University Institutional Review Board. This MRI dataset was originally reported by Guilmette et al. [23] in 1989 and has been implemented in a number of nasal particle deposition experiments [24], [25] and simulations [26], [27]. The multi-slice MRI scans were first segmented using MIMICS (Materialise, Ann Arbor, MI) into a 3-D model, which was further converted into a set of contours that defined the airways of interest. Based on these contours, an internal surface geometry was constructed in Gambit 2.4. The resulting model is intended to accurately represent the anatomy of the nasal airway with only minor surface smoothing. This model can either be manufactured into a solid cast by prototyping techniques for *in vitro* studies, or be meshed with high-quality computational elements for numerical analysis.

### Electrophoretic Olfactory Delivery Protocol

A simple device of such delivery protocol will consist of components with the following four functions: (a) aerosol generation (inhaler), (b) particle charging, (c) particle focusing, and (d) particle navigation control (Fig. 1b). More elaborate devices can add supports to stabilize the device relative to the human head. Particles are to be generated using available inhaler devices such as a nebulizer, pMDI, and dry powder inhaler [28]. The particles then pass through a charging chamber and acquire a given number of charges (usually positively charges) [29]. The positively charged particles subsequently enter the focusing chamber that has multiple slits [30]. The first slit has a high positive voltage and the last one has zero voltage. As the particles pass through the slits, the inward repulsive force drives the aerosol into a finely focused beam; meanwhile the forward repulsive force accelerates the particle beam to a certain exit speed (or inlet speed into the nostrils). The advantage of knowing the nasal inlet speed is that the particle trajectory can be predetermined for a given release point. Yet another advantage is that the speed of an aerosol could be much higher than the inhaled air speed so that it does not rely on inhalation maneuvers, making it suitable for seniors or patients with respiration difficulties. Once entering the nasal cavity, the particles are subjected to the electrophoretic force and begin to deviate from their original trajectories in a controlled manner (i.e., particle guidance). A precise control of the path deviations will guide the particles to the targeted olfactory receptor with minimal loss to the nasal valves and turbinates. In this study, we will limit our attention to the feasibility of electrophoretic guidance of neurological drug particles with electric potentials and charge levels that are safe to the human body.

### Fluid-Particle Transport Models

Flows in this study were assumed to be isothermal and incompressible. Steady inhalation and normal breathing conditions was assumed for all simulations. The mean inlet Reynolds number at the nostrils was around 1,413 [31]. The flow conditions inside the human nasal airways are predominately laminar flows. Therefore, the laminar flow model has been adopted in this study.

The trajectories of monodispersed particles with diameters (*d_p_*) were calculated on a Lagrangian basis by directly integrating an appropriate form of the particle transport equation. A smaller particle has a smaller inertia, a larger diffusion, and a larger influence from the electrophoretic force. The particles considered in this study were 0.5 µm in diameter because of their relatively small inertial and diffusive properties while maintaining a relatively large electrophoretic force. Aerosols of this size range have very low Stokes numbers (*St_k_* = *ρ_p_d_p_^2^UC_c_/18 µD_h_*<<1), where *ρ_p_* is the particle density (1.0 g/cm^3^), *C_c_* the Cunningham slip correction factor, *μ* the fluid viscosity, *U* the mean fluid velocity, and *D_h_* the hydraulic diameter of the nostril. The governing equation for spherical particle motion under these conditions can be expressed as [32],
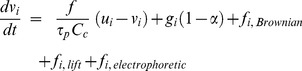
(1)where *v_i_* is the particle velocity, *u_i_* is the local fluid velocity, and *τ_p_* (i.e., *ρ_p_ d_p_* ^2^/18 *μ)* is the characteristic time required for a particle to respond to changes in the flow field. The drag factor *f*, which represents the ratio of the drag coefficient *C_D_* to Stokes drag, is based on the expression of Morsi and Alexander [33] for aerosols greater than 1 µm. Non-continuum slip effects on the drag of nanoparticles are accounted for using the Cunningham correction (*C*c) factor [13]. The gravity force was included with the gravity vector oriented in the vertical direction. The Saffman lift force was calculated for particles greater than 1 µm [34]. The effect of Brownian motion was considered for particles less than 1 µm [35]. One-way flow-particle coupling was implemented in this study because of the diluted concentration of drug aerosols. The computational fluid-particle dynamics model used in this study has been validated against existing experimental data in comparable airway models [36], [37]. The numerically determined deposition results agreed with experimental data within 5% for both sedentary (15 L/min) and light activity (30 L/min) inhalation conditions, indicating that the model hereof was adequate to capture the physical realism of interest.

### Electric Field and Electrophoretic Force

For the DC field, the electric potential, *U_DC_*, is obtained by solving Poisson’s equation,
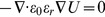
(2)where ε_0_ is the permittivity of free space (F/m) and ε_r_ is the relative permittivity of free space. The zero on the right hand side of the above equation indicates that the space charge density is negligible. For the AC field, the conservation of electric currents is solved to compute the AC potential, V [38]:
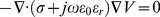
(3)where σ is the electrical conductivity and ω is the angular frequency (Hz). The total electric field is obtained from the superposition of the DC and AC fields considering that the equations for DC and AC fields are linear.

The electrophoretic force is computed by
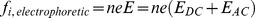
(4)


Where *n* is the dimensionless charge number and e is the elementary charge (*e* = 1.6×10^−19^ C), *E_DC_* and *E_AC_* are electric field intensity which are computed as follows.
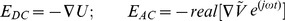
(5)


The symbol 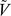
denotes that the AC electric potential is a complex value. In this study, only voltages less than 12 V were tested, which fell well within the human safety range (<50V) [39]. The corresponding operating parameters such as optimal particle size, particle charge, drug release velocity/position, and electrode layout/strength will be systematically explored, as described below.

The relation between the applied voltage parameters and particle path will be first explored in a quadrupole geometry. For given particle paths, the required voltage parameters will be calculated. These parameters will then be implemented in the two-dimensional (2-D) and 3-D nose models to calculate the particle trajectories. Considering the multiphysics involved and the prolonged computational expenses incurred, use of a 2-D model dramatically reduces computational times relative to 3-D modeling. It also allows us to reduce the model complexity without diminishing its integrity, gain understanding of fundamental physics underlying the transport process, and fine-tune the operating parameters to be used in the more intricate 3-D models. In this study, the influence of nasal airflow, electrode strength and drug release position will be studied on the olfactory deposition. The performance of the proposed delivery protocol will be evaluated by comparing particle transport and deposition between cases with and without electrophoretic guidance, as well as cases with and without particle focusing (pointed release).

### Numerical Method and Grid Sensitivity Analysis

To solve the metaphysics involved in this study, Comsol (Burlington, MA) was used to simulate the relevant multiphysics that include airflow, electric force, and particle tracing. Convergence of the flow field solution was assumed when the global mass residual was reduced by four orders of magnitude and the residual-reduction-rates for both mass and momentum were sufficiently small. The computational mesh was generated in Comsol in multi-domains with a physics-controlled manner. The nasal airway was discretized based on fluid physics, and the remaining domains (electrodes and open space) were based on general physics. To resolve the possible steep gradients of flow variables in the near-wall region, body-fitted mesh was also utilized. In light of the high complexity of nose morphology, corner mesh refinement was implemented throughout the model geometry. A grid sensitivity analysis was conducted by testing the effects of different mesh densities with approximately 0.4 million, 0.790 million, 1.1 million, and 2.0 million elements (degrees of freedoms). The predicted deposition rate was less than 1% when increasing mesh size from 1.1 million to 2.0 million. As a result, the final grid for reporting flow field and deposition conditions consisted of approximately 1.1 million cells.

## Results

### Particle Focusing and Guidance in a Quadrupole Model

**Figure 2:**
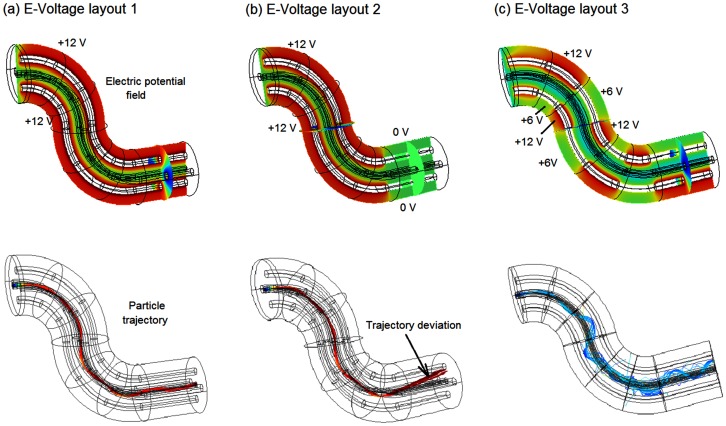
Electric field and particle trajectories within the quadrupole geometry with three electrode voltage layouts: (a) layout 1, (b) layout 2, and (c) layout 3.

The feasibility of electrophoretic focusing and guidance was first tested in a quadrupole geometry (Fig. 2), in which the trajectory of charged particles can be manipulated by varying the electrode arrangements. In this application, the applied voltage was 12 V and the particle size was 0.5 µm with 200 elementary charges. The inlet velocity of the airflow and charged particles was 0.1 m/s. The electric field and particle trajectories in the quadrupole geometry are shown in Fig. 2 for three test cases with different electrode layouts. In the first trial, we tested the sensitivity of charged nanoparticles to the electrophoretic guidance by varying the electric fields in both direction and strength. In doing so, eight groups of electrodes were arranged along the curve so that different voltage combinations can be specified. In Fig. 2a, the two opposite electrodes had the same voltage (12 V or −12 V). Similarly, a zero electric field was achieved along the curved centerline. The particles were concentrated within this field and at the same time were guided toward the exit, maneuvering through the curvatures by overcoming the inertial force.

**Figure 3:**
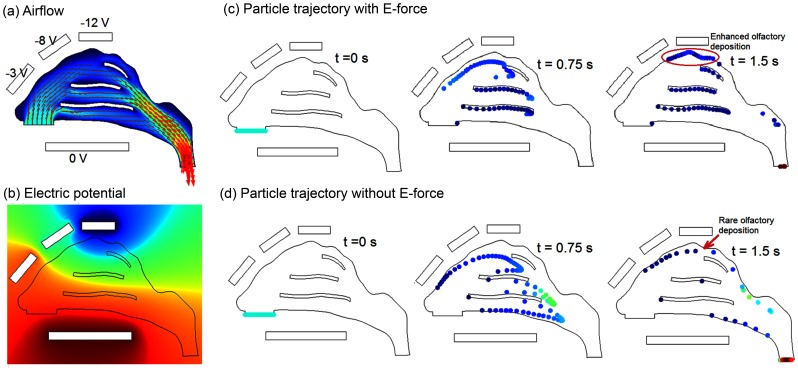
Dynamics of 2-D intranasal delivery: (a) airflow, (b) electric potential field, (c) particle trajectories with electrophoretic forces, and (d) particle trajectories without electrophoretic forces. In the electric field, the red color represents zero voltage and the blue color represents negative voltages.

In the second trial (Fig. 2b), we removed the voltage from the last two electrode groups to verify the indispensability of electrophoretic forces in particle focusing and navigation. As expected, without electrophoretic forces, particle trajectories departed from the centerline, indicating a break of force balance between the electrophoretic and inertial forces. A careful inspection of Figs. 3a&b further revealed this delicate force balance, which was manifested in the wavelets or fluctuations of particle trajectories along the curved centerline.

The third trial tested the capacity of electrophoretic forces in modifying particle trajectories by adjusting the electrode layout and/or strength (Fig. 2c). In this test, the two opposite electrodes in the same group had different voltages (12 V, 6 V), with the two neighboring groups alternating the voltage arrangement, as shown in Fig. 2c. It was observed that the particle trajectories were indeed modified, which largely followed the resultant zero-E field. This is desirable because it provides a promising tool in guiding the drug particles through convoluted nasal passages and navigating them away from the nasal turbinates. A possible disadvantage of this method is that the level particle focusing might be compromised, as shown by the more erratic and dispersive particle traces in Fig. 2c.

### Two-dimensional Nose Model

The feasibility of the new delivery protocol was further evaluated in a MRI-based 2-D nose model. Figure 3a shows the nasal airflow field, which is divided into three streams by the inferior and middle conchae (or turbinates). The upper stream is further split by the superior concha, with only a minor portion of the flow stream being distributed to the olfactory region (the uppermost location of the nasal cavity). Overall, the upper nasal cavity receives a small fraction of the inhaled airflow, indicating low particle transmission probability to this region. From Fig. 3a, the streamlines that start from the anterior nostril move towards the upper passage while those from the posterior nostril moves towards the nasal floor (i.e. inferior meatus). It is therefore conceivable that particles administered at the nostril tip have a higher possibility to reach the olfactory region.

To test the electrophoretic intranasal delivery protocol, four electrodes were arranged around the nose. The electric potentials were set to −3 V, −8 V, −12 V, and 0 V, respectively (Fig. 3a). The small voltage differential (3 V) in the nasal vestibule was to provide a slight driving force toward the upper nasal cavity while at the same time trying to avoid particle collisions with the vestibule. The voltage differential increased to 8 V after the nasal valve region to further guide particles toward the olfactory region. The voltage differential increased to 12 V directly above the olfactory region to attract the drug aerosols. Figure 3b displays the resulting electric potential field. The E-field is observed to change from nearly 0 V at the nostrils to nearly −12 V at the olfactory region.

Figures 3c and 3d compare the particle positions with and without electrophoretic forces at varying instants from their release at the nostrils. The particles considered are 0.5 µm in diameter with a density of 1 g/cm^3^ and a charge number of 200. Both airflow and particles enter the nostril at a speed of 0.1 m/s. The particle profiles without and with electrophoretic forces appear similar initially. The deviation becomes apparent thereafter (0.75 s) and continues to increase till 1.5 s. Due to the increasing upward electrophoretic force, a majority of the particles impinge on the three nasal conchae (or turbinates), and only a small portion of particles (∼14%) reach the olfactory region (Fig. 3d). In contrast, there is almost no deposition in the olfactory region without electrophoretic guidance (Fig. 3c).

**Figure 4:**
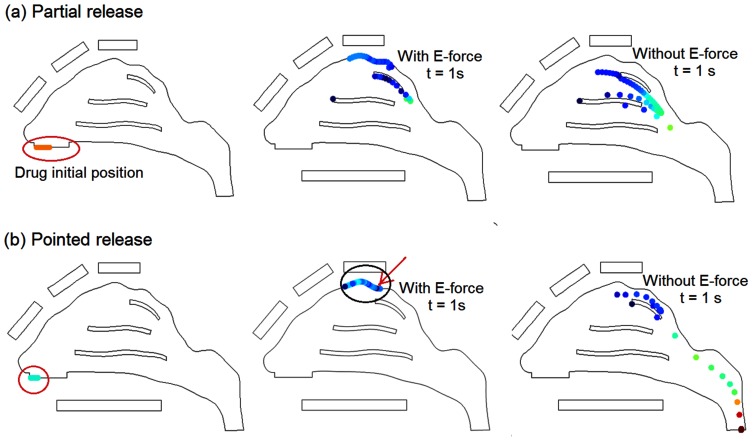
Particle trajectories with and without electric forces: (a) partial release; (b) pointed release.

Based on the observation that conchae depositions are predominately from particles that are released at the lower half of the nostril, it is natural to conjecture that by avoiding drug release from this region unwanted depositions in the nasal conchae will decrease. Figure 4a shows scenarios of administering drugs from the upper half of the nostril only (i.e., partial drug release), which significantly reduces the deposition in the middle concha and nearly eliminates the deposition in the inferior concha. In the case with electrophoretic guidance, partial release delivers about 45% of the drugs to the olfactory region, while the remainder (∼55%) depositing on the superior concha (Fig. 4a). To further reduce drug waste, we refined the drug release area so that all particles from that area could navigate through the nasal passages and reach the olfactory region. Figure 4b shows the particle dynamics one second after their release from a pointed region at the tip of the nostril. Nearly all electrophoretic-driven particles from this region (∼95%) deposit on the olfactory region. In contrast, only 0.77% of the particles released from this region end up in the olfactory region in the absence of electrophoretic forces.

**Figure 5:**
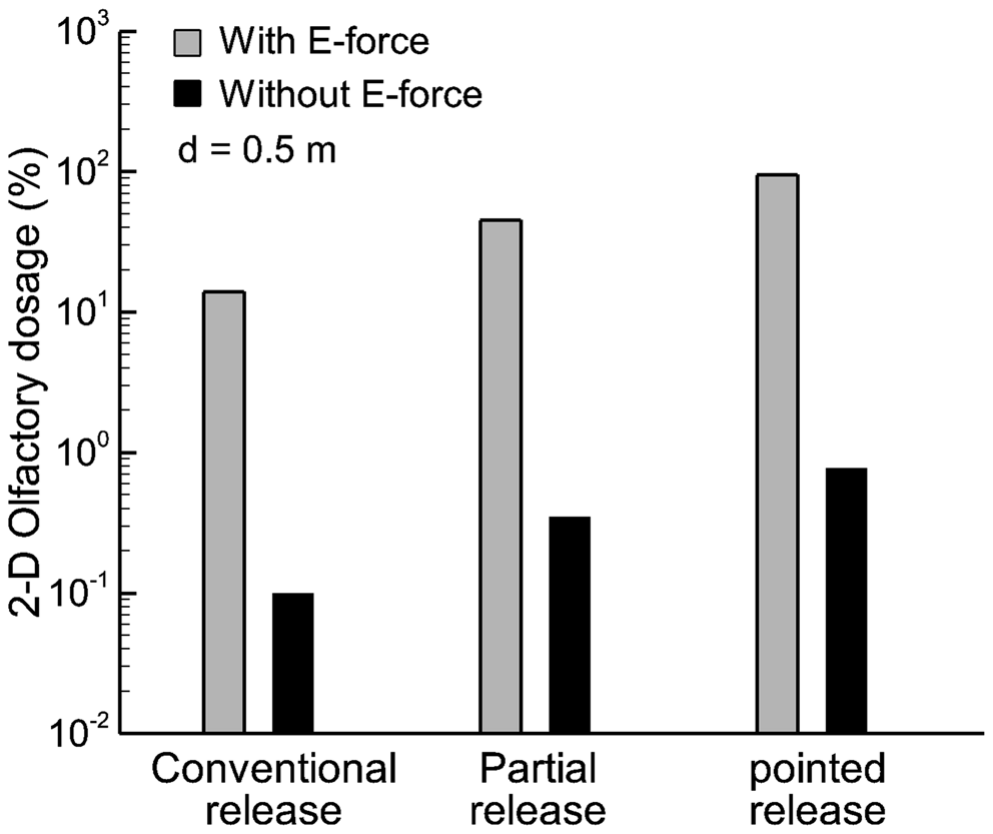
Comparison of olfactory dosages with and without electric forces in the 2-D model on a logarithmic scale. The olfactory dosages can be two orders of magnitude higher with applied E-forces than without.

The deposition rates of drug particles at the olfactory region (i.e., olfactory dosage) are shown in Fig. 5 using a logarithmic scale. From Fig. 5, applying electrophoretic forces remarkably enhances the olfactory dosage. In this pilot 2-D study, electrophoresis-guided delivery gives rise to olfactory dosages that are two orders of magnitude higher compared to drug deliveries without electrophoretic forces. Furthermore, releasing drugs into the upper half portion of the nostril (i.e., partial release) leads to an olfactory dosage two times higher than when releasing drugs over the entire nostril area (i.e., conventional release). The optimal release position with electrophoretic guidance is located at the top of nostrils, which delivers 95% drugs to the olfactory region. Considering the extremely low olfactory delivery efficiency (0.11–0.77%) with traditional approaches, this new protocol is highly advantageous in developing novel devices specifically for the nose-to-brain drug delivery.

### Three-dimensional Nose Model

The rationale underlying 2-D studies is that the motion of a particle can be largely assumed as planar and that the sideway motions are insignificant. This is reasonable when the purpose is to identify the appropriate operating parameters (electric potential and mass/charge ratio) to be used in 3-D scenarios. However, nasal airflows are fundamentally three dimensional, with significant secondary motions distributing the inhaled air/particles within the nose in a highly heterogeneous manner. Moreover, 2-D studies are inadequate when the electrode layout is sought to generate a 3-D electrical field for precise particle guidance.

**Figure 6:**
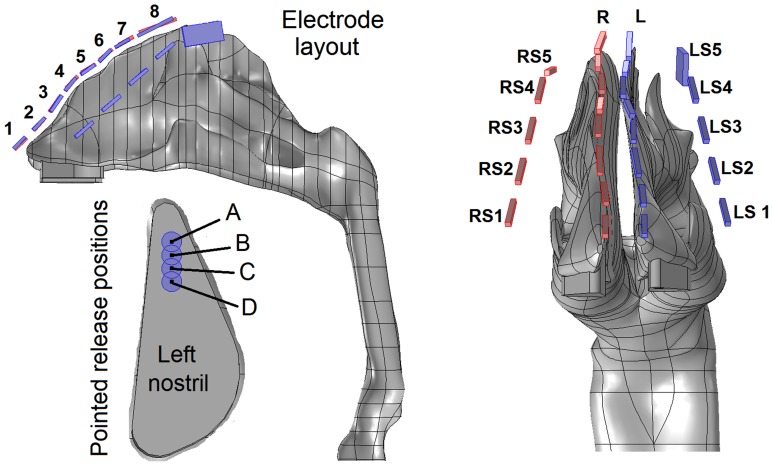
Diagram of electrode configuration and four particle-pointed-release positions. There are four groups of electrodes in tandem, with one group on each side of the nose and one group lining above each of the two nasal ridges. L: left; R: right; LS: left side; RS: right side.

Figure 6 shows the diagram of the electrode layout for the electrophoretic olfactory delivery. There are four rows of eletrodes, with two rows positioned along the ridges of the two nasal passges and the other two along the sides of the nose (Fig. 6). Approporiate electric poentials are specfied in order that the therapeutic agents administered at the nostrils can (a) escape the nasal valve/turbinate capture and (b) deposit in the olfactory region. Accordingly, two external force components are required: an upward force that drives particles toward the olfactory region by overcoming gravity and the downward drag force, and a lateral (x-direction) force that keeps the particles from depositing on the side walls of the conchae. The two top rows, which are negatively charged, provide the upward force, while the two side rows provide the lateral forces. In this study, medications are adminsitrated only at the left nostrils, and therefore only electrordes on the left top rows and the two side rows are used (Table 1). Furthermore, to evaluate the influence of different pointed release positions on olfactory dosing, a 1.5 mm-diameter catheter located at four locations (1 mm apart) in the left nostril will be tested (Fig. 6). The particles considered are assumed to be monodispersed and have a diameter of 0.5 µm, a density of 1 g/cm^3^, and carry 200 electric charges [13].

**Table 1:** Electric potentials on the electrodes of top left (L) and left side (LS) rows.

Voltage (V)	1	2	3	4	5	6	7	8
Left (L)	0	2	2	4	−4	−8	−12	−12
Left Side (LS)	1	1	3	0	−12	NA	NA	NA

**Figure 7:**
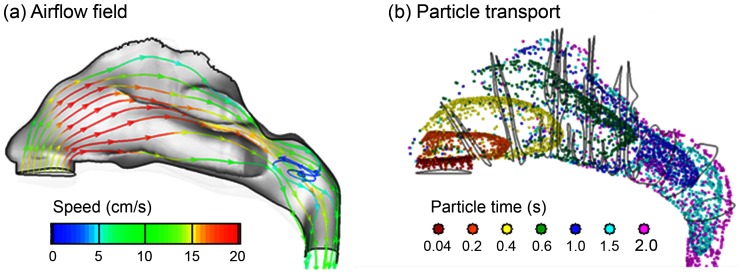
Inhalation airflow streamlines (a) and snapshots of particle transport (b) at different instants inside the nasal cavity.

Detailed knowledge of aerodynamics is crucial in predicting the behavior and fate of inhaled agents. The airflow fields and particle dynamics in the 3-D nasal airway are shown in Fig. 7 for an inhalation velocity of 0.1 m/s. Similar as the 2-D case, flows of high velocity magnitude occur in the middle portion of the medial passage, while the narrow fin-like meatus regions receive a small fraction of the airflow. The main flow changes its direction dramatically from the nostrils to the nasopharynx, forming a nearly 180° curvature. A recirculation zone is observed in the upper dorsal part of the nasopharynx as a result of sudden area expansion in both cross-sectional and effective flow areas (Fig. 7a). It is also observed that only the streamlines from the anterior nostril travel to the olfactory region, which move upward prior to the olfactory and downward after it.

**Figure 8:**
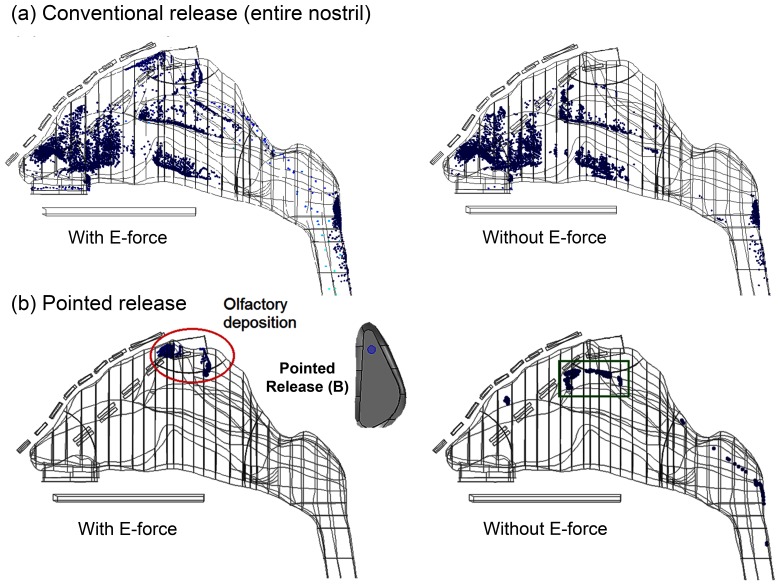
Comparison of deposition patterns with and without electrophoretic forces for particles released (a) over the entire area of the left nostril, and (b) at point B of the left nostril.

For the breathing condition considered in this study (0.1 m/s), it takes 0.6–1 seconds after administration for the particles to reach the olfactory region (Fig. 7b). Because of the dramatic airway bend from the nostrils to the nasopharynx, a high-concentration of particles constantly adjust their directions following the mean streamline curvature of inhaled airflows. Faster transport and deeper penetration of aerosols are apparent in the medial passages while slow-moving particles are found near the airway walls. Only a small portion is observed to deposit in the superior meatus, with even less particles reaching the upmost olfactory region. The surface deposition of inhaled aerosols is accordingly highly heterogeneous, as illustrated in the surface deposition (Fig. 8a).

A comparison of deposition patterns with and without electrophoretic forces is illustrated in Fig. 8 for particles of 0.5 µm diameter and 200 electric charges. In both cases, 10,000 particles are released evenly over the entire area of the left nostril. With a proper electric potential layout (Table 1), a noticeable percentage (3.7%) of administered medications reaches the targeted olfactory region (Fig. 8a, left panel). In contrast, the possibility of olfactory deposition is negligible (0.06%) in absence of electrophoretic forces (Fig. 8a, right panel).

To further increase the olfactory delivery efficiency, medications are administered at one small point (Point B in Fig. 8b) in the anterior nostril to help escape the nasal valve/turbinate capture. In both cases, drug loss to the nasal valve and turbinates are remarkably diminished. More importantly, with electrophoretic guidance, a majority (92%) of therapeutic agents made their way to the olfactory (red ellipse) that would have otherwise deposited in the superior turbinate and nasopharynx (Fig. 8b, left panel vs. right panel). A careful inspection of the deposition in the superior turbinate (green square) in the right panel of Fig. 8b reveals that particles are deposited at the outer side (turbinate surface) in contrast to the inner septum wall. To reduce deposition here, a lateral force component is needed to repel these particles from approaching the outer wall.

**Figure 9:**
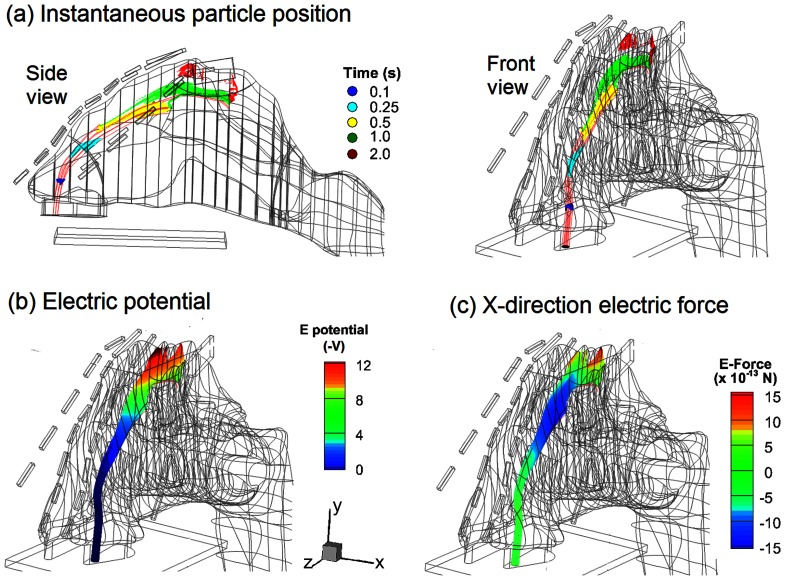
Snapshots of particle locations (a), electric potential (b) and electrophoretic force in x direction (c) in the left nasal passage for particles released at point B.

The mechanism underlying the electrophoretic olfactory delivery is illustrated in Fig. 9, which shows the deflection of charged particles from the airflow streamlines, the electric potential field, and the x-direction (lateral) component of the resultant electric force. The deflection becomes apparent initially in the region beneath the olfactory. As discussed before, it takes 0.6–1.0 s for particles to travel from the nostril to this region, mainly due to the aerodynamic forces. After that, the upward electrophoretic force becomes dominant, which alters the particle trajectory and orientates them towards the olfactory. This process is slow and can take 1–2 seconds, as indicated by the color migration from green to red in Fig. 9a.

The external electric potential field and electrophoretic force experienced by drug particles are shown in Figs. 9b&c. To successfully maneuver the particles through the convoluted nasal passage, the electric potential must vary in both magnitude and direction along the particle path, depending on the path deflections required to escape being captured by the nasal valve or turbinates. In this case, particle motion can be divided into three stages: the aerodynamically controlled stage (0–0.5 s) that transports particles from the nostril to the superior meatus, the second stage where both drag and electric forces are important (0.5–1 s), and the third stage (1–2 s) where the particles are diverted to the olfactory by electrophoretic attractions. In the second stage, a lateral electric force is exerted on the charge particles that points toward the septum (negative x-direction in Fig. 9c) and helps to divert the particles from depositing onto the turbinate.

**Figure 10:**
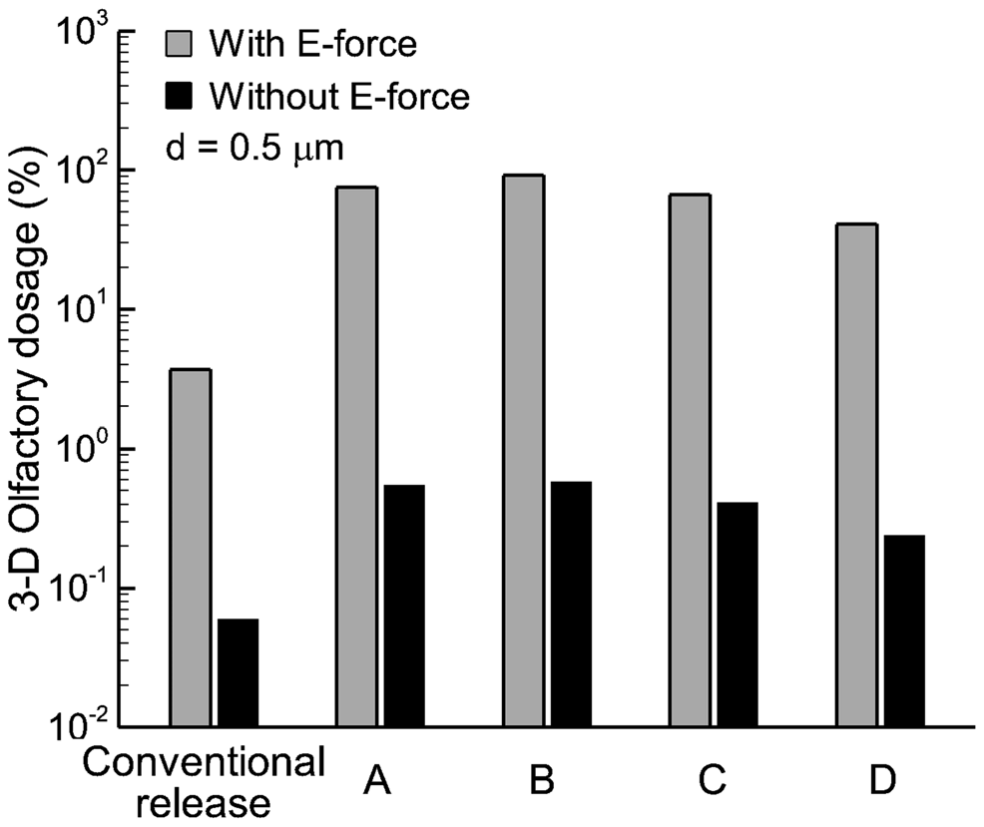
Comparison of olfactory dosages with and without electric forces in the 3-D model on a logarithmic scale. The olfactory dosages can be two orders of magnitude higher with applied E-forces than without.

Figure 10 compares the predicted olfactory delivery efficiency with and without electrophoretic forces using a logarithmic scale. Overall, the numerical predictions in the 3-D model corroborate with the 2-D results that electrophoretic forces can significantly enhance olfactory delivery. With appropriate E-forces, the olfactory deposition rates can be two orders of magnitude higher than that without electrophoretic control. Furthermore, administering therapeutic agents at a specific location (i.e., pointed drug release) further enhances the olfactory delivery over the conventional practice of administering drugs over the entire area of the nostril (conventional release). It is also noted that the olfactory delivery efficiency is highly sensitive to the pointed release position. For a given electric field and its optimal release point (point B in this study), the olfactory dosage decreases from 92% at point B to 75% by shifting the release position only 1 mm forward to point A, reduces to 67% by 1 mm backward to point C, and to 41% by 2 mm backward to point D. Charged particle transport through an airflow and electric field is a dynamic process. The trajectory of such a particle is a result of the interplay between aerodynamic and electric forces. Moreover, the relationship between the electrophoretic deflection and electric potential is nonlinear (second-order derivative in nature) and is spatially dependent (local velocity and electric field). Considering these interacting factors, it is not surprising that the olfactory dosing will exhibit a high sensitivity to different release locations.

## Discussion and Summary

In this pilot study, we demonstrated the feasibility of intranasal drug delivery to the olfactory region with electrophoretic guidance in an image-based human nose model. Effective olfactory dosing is the crucial first step in direct nose-to-brain delivery, the feasibility of which rests on two factors: effective dosage to the central nervous system (CNS) and no obvious systemic side effects. In this regard, the optimal olfactory dosage (92%) predicted in this study appears very promising. The highest voltage applied around the nose is only 12 V, which is safe to the human body and can be easily achieved using a 12 V battery.

The complexity of the nasal cavity precludes effective intranasal drug delivery. Most inhaled particles will impinge on the intranasal walls and be filtered out of the respiratory tract. This explains how the human nose functions as an effective filter and is considered our first defense line. However, this same defense has for decades baffled efforts in searching for effective methods to deliver medications via the nasal route to the lungs, olfactory epithelium, or paranasal sinuses. Results of this study showed that, with an appropriate electric field, a particle’s trajectory can be precisely controlled so as not to intercept the airway surface before reaching the olfactory region. Success of such control rests on the accurate knowledge of two factors: (1) the original course/trajectory of inhaled particles, and (2) the course deflection required to escape nasal valve/turbinate filtrations. The first factor can be readily identified with patient-specific image-CFD modeling to determine the necessary deflections (the second factor). The amount of deflection for a given particle depends on the concurrent actions of electric and aerodynamic forces, which are further determined by the electric potential field and the instantaneous particle speed (residence time). Accordingly, if the particle mass/charge ratio and inhalation rate are known, the electric field required for trajectory defection can be calculated, which can be used to calculate the electrode layout and strength. It is noted that there should be deflections in more than one direction to ensure effective olfactory dosages, as shown in Fig. 1.

The novel aspect of this study is that charged nanoparticles can be precisely controlled using appropriate electrophoretic forces. This protocol provides multiple advantages that are lacking in current inhaler devices. For example, electrophoresis-guided drug particles can be contact-free from the airway wall, which significantly reduces drug waste in the nasal valve/conchae and increases the olfactory dosage. As a result, it has the potential to overcome the nose-to-brain bottleneck posed by extremely low olfactory depositions [9]. Drug particles can be free of ferric magnetic materials, therefore minimizing side effects from metallic accumulations [40], [41]. Furthermore, electrophoresis-guided delivery does not rely on inhalation conditions, which makes it suitable for seniors or patients with respiratory distress who have difficulties in exercising required breathing maneuvers [42], [43], [44]. The proposed platform can also be easily adapted for target drug delivery at respiratory sites other than the olfactory region by modifying electrode layouts and voltage gradients.

Clinically, the electrophoretic guidance system is envisioned to consist of one zero-voltage electrode positioned within the mouth along with four rows of negative electrodes positioned outside the nose. Each row will include multiple electrodes where different voltages are applied to create a desired electric field in the nose. A head brace can be used to stabilize the electrodes relative to the head so that there is no direct contact between the electrodes and human body. In this proposed protocol, a patient-specific image-CFD modeling is necessary to determine the required deflections. The cost incurred by MRI scans and CFD modeling could be a potential barrier to its applications in human population.

Inflammation of the nasal mucous membrane due to allergic rhinitis or rhinosinusitis leads to narrowing and obstruction of the already narrow nasal passages. It will conceivably be more challenging to guide drug particles through these passages without surface contact loss, which requires more precise control on particle trajectories to achieve satisfactory delivery efficiency. Modifications to the electric potential gradients are necessary to correct the altered particle trajectory deflections. For severely obstructed scenarios, reduced olfactory delivery efficiencies are expected. To remedy this setback, vasoconstrictors such as xylometazoline should be given first to patients with rhinitis and rhinosinusitis to alleviate the nasal inflammation before administering medications. Generally, the nasal mucus carries a negative charge due to sodium and chloride movements across the membrane (i.e., −5 to −30 mV for normal subjects and −40 to −80 mV for Cystic Fibrosis patients) [45]. However, the potential effect of this charge on particle trajectories is expected to be negligible, considering that it has a much smaller magnitude relative to the applied electric voltages.

Limitations of this study include the assumption of steady flows, rigid airway walls, limited electrode layouts/operating parameters, and neglect of facial topology. In this study, special considerations had been taken to minimize the flow effects. First, a very low inhalation rate (0.1 L/min) was adopted to reduce the particle inertial effect and increase the particle reaction time to electrophoretic controls. Second, this proposed delivery protocol mainly depends on the electrophoretic forces and therefore has less dependence on breathing conditions. Practically, the influence of transient breathings can be further minimized by instructing the patient to inhale steadily and by activating the drug release only during this quasi-steady inhalation phase. Considering the non-linear relationship between particle trajectory and electrophoretic force, identifying the optimal values of the electric voltages is basically an art mixed with science, necessitating numerous trials to find the best combination. This complexity is further compounded by the need for precise path control that navigates the particles through the convoluted nasal passages without loss to the wall. Facial topology may also affect the layout of electrodes. Each limitation influences the realism of the model predictions and should be addressed in future studies. Additionally, this study is limited to the nose models based on one subject and therefore does not consider the inter-subject variability. Future numerical studies with more image-based nose models, as well as complementary experimental studies are necessary to advance our knowledge of the feasibility and efficiency of this new drug delivery strategy.

In conclusion, this study systematically evaluated the feasibility and effectiveness of targeted drug delivery with electrophoretic forces in both idealized and realistic nose models. Specifically, the influences of electric fields and drug-release positions were examined in controlling the particle motion toward the olfactory region. Results of this pilot study have implications for the development of effective intranasal drug delivery devices that target the olfactory epithelium. Specific findings of this study are:

1. It is feasible to focus and guide nanoparticles with electric voltages that are safe to the human body (<12 V).
2. Applying electrophoretic forces can significantly increase the olfactory deposition of nanoparticles with diameters of 0.5 µm and below.
3. Electrophoretic olfactory delivery exhibits high sensitivity to drug release positions.
4. With appropriate electrophoretic guidance and selective drug release, an olfactory delivery efficiency of more than 90% can be achieved.
